# 225. Dalbavancin Usage in Patients Who Inject Drugs (PWID)

**DOI:** 10.1093/ofid/ofad500.298

**Published:** 2023-11-27

**Authors:** Kaya Patel, Peter Nakagami, Kazumi Morita, Jason Gallagher, Sara K Schultz

**Affiliations:** Temple University Hospital, Philadelphia, Pennsylvania; Temple University Hospital, Philadelphia, Pennsylvania; Temple University Hospital, Philadelphia, Pennsylvania; Temple University, Philadelphia, Pennsylvania; Temple University Hospital, Philadelphia, Pennsylvania

## Abstract

**Background:**

Dalbavancin is a lipoglycopeptide antibiotic approved for the treatment of acute bacterial skin and skin structure infections (ABSSSI) secondary to gram-positive organisms. Given its long half-life, there has been increasing off-label use for other indications: osteomyelitis, joint infections, and bacteremia. One application where it can be effective is in PWID due to high rates of incomplete IV therapy. Herein we describe characteristics of PWID patients that receive dalbavancin and outcomes including recurrence of infection.

**Methods:**

We conducted a retrospective chart review of all patients receiving dalbavancin at our large academic medical center in Philadelphia between Nov 1, 2019, to October 31, 2022. Charts were reviewed in Epic with data collection in REDCap.

**Results:**

Fifty-two patients with substance use disorder with injection behavior that received dalbavancin were reviewed. Forty-seven (90%) of these patients had unstable housing. Patients were recommended dalbavancin due to concerns for adherence to prolonged oral regimen (31, 60%) or to avoid lengthy hospitalization which did not align with patient’s goals of care (43, 83%). Patient directed discharges occurred in 20 (38%) patients. Indications included bone/joint infections (25, 48%), bone/joint infections involving prosthesis (3, 6%), bacteremia (9, 17%), endocarditis (3, 6%) and ABSSSI (24, 46%). Twenty-one (40%) patients who received dalbavancin avoided a full IV antibiotic course. Forty-eight (92%) patients received effective antibiotics prior to dalbavancin (average of 6.2 days). Forty-three (83%) patients received a full dalbavancin course, of which 2 (5%) patients were readmitted for relapsed infection in 90 days. Nine (17%) patients received partial dalbavancin treatment courses, of which 6 (66%) had 90-day readmissions for relapsed infection. (Table 1).

**Figure:**
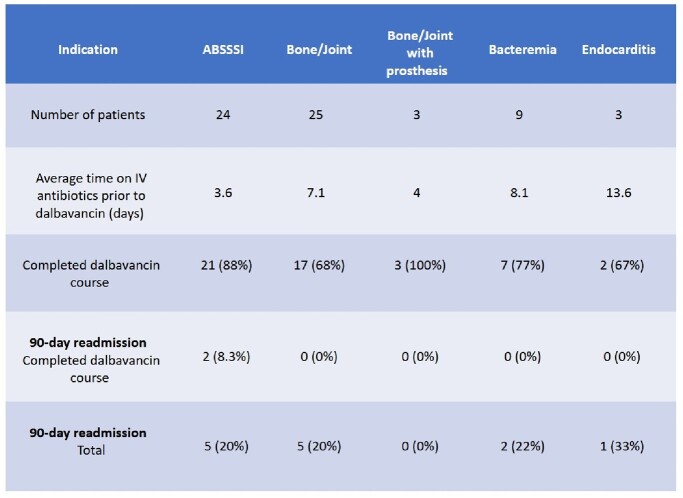

**Conclusion:**

At our institution, dalbavancin was used to treat infections beyond ABSSSI, in a population of predominantly PWID. We show low rates of 90-day readmission for recurrent infection, including patients with bacteremia and endocarditis, following full courses of dalbavancin. Dalbavancin is a viable alternative to lengthy oral or IV antibiotic regimens in patients that would otherwise not receive full treatment courses.

**Disclosures:**

**Jason Gallagher, PharmD**, Entasis: Advisor/Consultant|Merck: Advisor/Consultant|Merck: Grant/Research Support|Qpex: Advisor/Consultant|Shionogi: Advisor/Consultant|Spero: Advisor/Consultant **Sara K. Schultz, MD FACP FIDSA**, AbbVie: Advisor/Consultant

